# *Candidatus* Neoehrlichia mikurensis in rodents in an area with sympatric existence of the hard ticks *Ixodes ricinus* and *Dermacentor reticulatus*, Germany

**DOI:** 10.1186/1756-3305-5-285

**Published:** 2012-12-07

**Authors:** Cornelia Silaghi, Dietlinde Woll, Monia Mahling, Kurt Pfister, Martin Pfeffer

**Affiliations:** 1 Comparative Tropical Medicine and Parasitology, Ludwig-Maximilians-Universität München, Leopoldstr. 5, D-80802, Munich, Germany; 2 Institute of Animal Hygiene and Veterinary Public Health, University of Leipzig, Leipzig, Germany; 3 Statistical Consulting Unit, Department of Statistics, Ludwig-Maximilians-Universität München, Munich, Germany

**Keywords:** *Candidatus* Neoehrlichia mikurensis, Bank vole, Yellow-necked mouse, *Ixodes ricinus*, *Dermacentor reticulatus*, Recreational area, Host survey, Vector-host relation

## Abstract

**Background:**

*Candidatus* Neoehrlichia mikurensis (CNM) has been described in the hard tick *Ixodes ricinus* and rodents as well as in some severe cases of human disease. The aims of this study were to identify DNA of CNM in small mammals, the ticks parasitizing them and questing ticks in areas with sympatric existence of *Ixodes ricinus* and *Dermacentor reticulatus* in Germany.

**Methods:**

Blood, transudate and organ samples (spleen, kidney, liver, skin) of 91 small mammals and host-attached ticks from altogether 50 small mammals as well as questing *I. ricinus* ticks (n=782) were screened with a real-time PCR for DNA of CNM.

**Results:**

52.7% of the small mammals were positive for CNM-DNA. The majority of the infected animals were yellow-necked mice (*Apodemus flavicollis*) and bank voles (*Myodes glareolus*). Small mammals with tick infestation were more often infected with CNM than small mammals without ticks. Compared with the prevalence of ~25% in the questing *I. ricinus* ticks, twice the prevalence in the rodents provides evidence for their role as reservoir hosts for CNM.

**Conclusion:**

The high prevalence of this pathogen in the investigated areas in both rodents and ticks points towards the need for more specific investigation on its role as a human pathogen.

## Background

Rodents are reservoir hosts for several zoonotic and emerging pathogens [1]. Furthermore, rodents and other small mammals serve as main feeding and maintenance hosts for the developmental stages of various tick species. They play an important role in the endemic cycles of tick-borne pathogens by being reservoir hosts for those as well as being drivers for the tick population itself (e.g. the tick-borne encephalitis-virus or *Babesia microti*) [2-4]. Thus, the health of human beings can be seriously impaired by contact with rodents or ticks, which have previously fed on them. Ticks found on small mammals can be either exophilic or endophilic. Exophilic species such as the anthrophilic *Ixodes ricinus* may act as bridge vectors between large mammalian species including humans and small mammals [5]. The developmental stages of *Dermacentor reticulatus* or endophilic species (e.g. *I. trianguliceps*) are more specialised regarding their hosts and may provide stable niche cycles for pathogens such as *Anaplasma phagocytophilum* and *B. microti*[6-8]. A new pathogen belonging to the α-proteobacteria (family *Anaplasmataceae*) was detected in the late 1990s in *I. ricinus* in the Netherlands and Italy as well as in a Norway rat (*Rattus norvegicus*) in China and was initially called *Ehrlichia*-like (or *Schotti variant*, *E. walkerii*, *Rattus*-strain) due to a diverging *16S rRNA* gene sequence [9-11]. Further findings in rats and *I. ovatus* ticks in Japan and the passaging of the agent in laboratory rats led to its description as the new species *Candidatus* Neoehrlichia mikurensis (CNM) in 2004 [12]. Although in parts genetically characterized, it was not possible till the present time to isolate the bacterium *in vitro,* thus formally remaining the taxonomical status as *Candidatus*. A recent study comparing *16S rRNA* and *groEL* gene sequences confirmed the identity or very close relationship of sequences from the initial findings of the *Ehrlichia*-like organisms with the new species [13]. This emerging zoonotic intracellular tick-borne pathogen forms a separate cluster in the family *Anaplasmataceae* together with the North American *Cand*. N. lotoris, which has been detected in raccoons [14-16]. CNM has been shown to be a human pathogen as it has been identified in the blood of febrile human patients in Germany, Sweden, Switzerland and the Czech Republic, and a dog in Germany. Most of these patients were immunocompromised due to splenectomy or immunosuppressive therapy and the reported diseases were severe, including hemorraghic events, recurrent fever of up to 8 months and even lethal outcome [14,17-21]. *I. ricinus* is most likely the vector for CNM in Europe, but the reservoir host is not fully known; however, rodents have been suggested [13]. Several studies have identified DNA of CNM in questing or host-attached *I. ricinus* in Europe including Germany [11,13,22,23]. However, potential reservoir hosts have thus far not been examined in Germany. Accordingly, in this study the occurrence of DNA of CNM was identified in small mammals, the ticks parasitizing them and questing ticks in recreational areas with co-existing *I. ricinus* and *D. reticulatus* populations in Germany. The aims were (i) to identify their infection rates, (ii) to determine the suitable screening material in rodents for epidemiological studies and (iii) to assess the co-infections with other pathogens in these areas where several pathogens have been found in rodents and ticks before [24,25].

## Methods

Small mammals were trapped and euthanized in a metropolitan area of Leipzig from August 2010 to November 2011 (Permission-No 36.45.12/4/10-026 of the city of Leipzig). All ticks found on the small mammals were collected. Questing ticks collected during 2008 and 2009 with the flagging method in Leipzig (Saxony), near Munich (Bavaria) and the Saarland were also available for this study. For the latter two sites no rodents were trapped. The sampling sites, results of the tick collection, procedures of the rodent trapping, rodent species trapped, necropsy, and DNA extraction from animal tissues, host-attached and questing ticks have been described in detail previously [25]. Additionally to the 80 previously described animals, 5 yellow-necked mice (*Apodemus flavicollis*) and 6 bank voles (*Myodes glareolus*) were available from November 2011. Blood (n=40), transudate (n=91) and organ samples [spleen (n=90), kidney (n=89), liver (n=90), skin (n=91)] of 91 small mammals, as well as all host-attached ticks (n=365; 234 *I. ricinus*, 117 *D. reticulatus*, 1 *I. trianguliceps,* 11 *Ixodes* spp., 1 *Dermacentor* spp., 1 without species identification due to damage) from altogether 50 small mammals, and questing *I. ricinus* ticks (n=782, in a total of 730 samples, as some nymphs were pooled) were screened for DNA of CNM. For 51 of the small mammals, no blood could be drawn prior to necropsy, for 2 animals no kidney sample, and for one animal each no spleen and no liver sample were available.

A real-time PCR previously published and targeting the *groEL* gene was used for the detection of CNM-DNA with modifications [13]. The reaction was carried out in an AB7500fast (Applied Biosystems, Germany) in a total volume of 20μl using 10μl of the Universal fast TaqMan Master Mix (Applied Biosystems), 1μl of molecular grade water, the following three primers (all 10μM; 1.8μl NMikGroEL-F2 5’-CCTTGAAAATATAGCAAGATCAGGTAG-3’, 0.9μl NMikGroEL rev1 5’-CCACCACGTAACTTATTTAGCACTAAAG-3’ and 0.9μl NMikGroEL rev2 5’-CCACCACGTAACTTATTTAGTACTAAAG-3’) and 0.4μl of the probe NMikGroEL-P2a 5’-FAM-CCTCTACTAATTATTGCTGAAGATGTAGAAGGTGAAGC-BHQ1-3’. 5μl of DNA-template was added to each reaction. The cycling conditions were 95°C for 20sec followed by 40 cycles (95°C for 3sec; 60°C for 30sec). To each reaction, a negative control (molecular grade water) and a positive control (DNA of CNM from naturally infected *I. ricinus*, confirmed by sequencing) were added.

Data on the occurrence of *A. phagocytophilum*, *Babesia* spp. and *Rickettsia* spp. in questing adult *I. ricinus* as well as of the small mammals and their ticks with *A. phagocytophilum* and *Babesia* spp. was available from previous investigations in the same animals and ticks. Hence, co-infections with CNM were analyzed [24,25].

### Statistical analysis

Exact confidence intervals of the prevalences of small mammals and questing ticks (CI 95%) were computed with the Clopper and Pearson method. For the questing ticks, differences in the infection rates with CNM between sex of ticks, collection sites and region were computed with logistic regression model using R 2.13.1 as described [25] (R Developmental Core Team 2011 [26]). Positive detection rates were compared among all groups (sex, sites, regions) by use of a procedure for simultaneous tests for general linear hypotheses in parametric models, which adjusts for multiplicity and controls the overall type I error rate [27]. The latter approach was also used for the calculation of differences in co-infection rates. P-values <0.05 were regarded as statistically significant. No modelling was attempted for the infestations of small mammals with ticks due to the irregular intervals of trapping.

## Results

### Small mammals

Altogether 48 out of 91 (52.7%; 95%CI: 42-63%) small mammals had detectable CNM-DNA in one or more of their organs or body fluids. 24 out of 37 yellow-necked mice (64.9%; 95%CI: 47.5-79.8%) and 23 out of 42 bank voles (54.8%; 95%CI: 38.7-70.2%) as well as 1 out of 3 striped field mice (*A. agrarius*) (33.3%; 95%CI: 0.8-90.6%) were positive. None of the insectivore species was positive. Most animals were positive in the spleen and the kidney (Table 1). A total of 19 out of 40 blood samples (47.5%) were positive. Animals that tested positive in the blood also tested positive in transudate, kidney, spleen and liver, with the exception that 4 of those animals were negative in the liver. The positive striped field mouse (out of three), however, tested negative in both blood and transudate, but was found to contain CNM-DNA in both spleen and kidney. One bank vole was positive exclusively in the blood. However, 6 animals positive in other organs were negative in the blood samples. One bank vole was positive in the skin sample and this animal was also positive in all other samples whereas all other animals were negative in the skin samples. One yellow-necked mouse was positive only in the kidney.

**Table 1:** PCR results for detection of DNA of *Candidatus* Neoehrlichia mikurensis in 91 small mammals from recreational areas in Leipzig (2010–2011)

			**Blood**			**Transudate**			**Spleen**			**Kidney**			**Liver**			**Skin**		
**Small mammalian species**	**Total number of animals**	**Pos, any**^**a**^	**Pos**	**Total**	**(%)**	**Pos**	**Total**	**(%)**	**Pos**	**Total**	**(%)**	**Pos**	**Total**	**(%)**	**Pos**	**Total**	**(%)**	**Pos**	**Total**	**(%)**
***Apodemus flavicollis*Yellow-necked mouse**	37	24	12	18	(66.6)	20	37	(54.1)	23	37	(62.2)	23	36	(63.9)	16	36	(44.4)	0	37	(0)
***Apodemus agrarius*Striped field mouse**	3	1	0	1	(0)	0	3	(0)	1	3	(33.3)	1	3	(33.3)	0	3	(0)	0	3	(0)
***Myodes glareolus*Bank vole**	42	23	7	21	(33.3)	19	42	(45.2)	22	42	(52.4)	21	41	(51.2)	18	42	(42.9)	1	42	(2.0)
***Arvicola amphibius*European water vole**	3	0	n.a.			0	3	(0)	0	3	(0)	0	3	(0)	0	3	(0)	0	3	(0)
***Talpa europaea*Common mole**	1	0	n.a.			0	1	(0)	0	1	(0)	0	1	(0)	0	1	(0)	0	1	(0)
***Crocidura russula*Greater white-toothed shrew**	4	0	n.a.			0	4	(0)	0	3	(0)	0	4	(0)	0	4	(0)	0	4	(0)
***Sorex araneus*Common shrew**	1	0	n.a.			0	1	(0)	0	1	(0)	0	1	(0)	0	1	(0)	0	1	(0)
**Total**	**91**	**48**	**19**	**40**	**(47.5)**	**39**	**91**	**(42.9)**	**46**	**90**	**(51.1)**	**45**	**89**	**(50.6)**	**34**	**90**	**(37.8)**	**1**	**91**	**(1.1)**

^a^ positive in any organ; pos., positive; n.a., not available.

Looking at the trapping months, 0 out of 8 animals (0%; 95%CI: 0–36.9%) caught from March to May, 13 out of 25 (52.0%; 95%CI: 31.3-72.2%) caught in June, 25 out of 33 (75.8%; 95%CI: 57.7-88.9%) in August, 6 out of 14 (42.9%; 95%CI: 17.7-71.1%) in October, and 4 out of 11 (36.4%; 95%CI: 10.9-69.2%) in November were positive.

### Ticks on small mammals

Small mammals with tick infestation [any tick species: 31 out of 53 (58.5%; 95%CI: 44.1-71.9%); *I. ricinus* infestation: 30 out of 49 (61.2%; 95%CI: 46.2-74.8%)] were more often infected with CNM than small mammals without ticks (17 out of 38, 44.7%; 95%CI: 28.6-61.7%). Altogether 27 ticks from 12 rodents were positive. Those were 15 out of 234 (6.4%) *I. ricinus* (9 larvae, 6 nymphs), 9 out of 117 (7.7%) *D. reticulatus* (all nymphs), 2 *Ixodes* spp. larvae and 1 larva for which species identification was not possible. The positive *I. ricinus*, 2 *Ixodes* spp. and the unidentified larva were from 11 individual mammals (5 yellow-necked mice, 5 bank voles, 1 striped field mouse) and of those animals, altogether 90.9% (10/11) were positive for CNM and of those 5 in the blood (for 4 no blood was available). The positive *D. reticulatus* were collected from 3 bank voles and all (3/3) of them were positive, also in blood.

### Questing ticks

A total of 539 *I. ricinus* from recreational areas in Leipzig, 128 *I. ricinus* from Bavaria and 115 *I. ricinus* from the Saarland were screened for the presence of CNM*-*DNA.

Altogether, 24.2 – 26.6% *I. ricinus* were positive for DNA of CNM. The range of infection rate is the minimal and maximal infection rate (MinIR, MaxIR), assuming that either only one or all of the nymphs in a pool were positive: 89/297 (30%; 95%CI: 24.8-35.5%) of the females, 75/313 (24%; 95%CI: 19.4-29.1%) of the males and 25-44/172 (19 individuals and 6 pools) of the nymphs (MinIR: 17.4%, 95%CI: 11.6-24.6% and MaxIR: 29.2%, 95%CI: 21.9-37.3%) were positive. Details for the individual sites and regions are shown in Table 2. Comparing regions pairwise with a linear hypothesis, significantly fewer ticks were positive in Bavaria than in Leipzig (p<0.001), but in the Saarland, significantly more ticks were infected than in Leipzig (p<0.001). Logistic regression models were used to assess the effects of sex/developmental stage and site and sex/developmental stage and region, respectively. A pairwise comparison between the major collection sites in Leipzig (E, H, I) revealed statistically significant differences between sites E and H (p<0.001) and H and I (p=0.01). There were no statistically significant differences between male and female ticks, but between females and nymphs (p<0.001) and males and nymphs (p=0.01).

**Table 2:** Infection rates with *Candidatus* Neoehrlichia mikurensis in questing *Ixodes ricinus* ticks from recreational areas in Bavaria, the Saarland and in and around the city of Leipzig, Saxony

**Region**		**Female**			**Male**			**Nymph**			**Total**		
		**Pos**	**Total**	**(%)**	**Pos**	**Total**	**(%)**	**Pos**	**Total**	**(%)**^**a**^	**Pos**	**Total**	**(%)**^**a**^
Leipzig		**67**	**212**	**(31.6)**	**52**	**219**	**(23.7)**	**20-28**	**108**	**(18.5-25.9)**	**139-147**	**539**	**(25.8-27.3)**
	Site												
	E	7	45	(15.6)	8	56	(14.3)	3	6	(50)	**18**	**107**	**(16.8)**
	F	2	6	(33.3)	1	4	(25.0)	1-5	10	(10–50)	**4-9**	**20**	**(20.0-45.0)**
	G	0	6	(0)	0	2	(0)	0	13	(0)	**0**	**21**	**(0)**
	H	35	74	(47.3)	18	53	(34.0)	10-14	45	(22.2-31.1)	**63-67**	**172**	**(36.6-39.0)**
	I	23	81	(28.4)	25	104	(24.0)	6	34	(17.6)	**54**	**219**	**(24.7)**
Bavaria		5	42	(11.9)	5	58	(8.6)	1	28	(3.6)	**11**	**128**	**(8.6)**
Saarland		17	43	(39.5)	18	36	(50.0)	4-12	36	(11.1-33.3)	**39-47**	**115**	**(33.9-40.1)**
**All regions**		**89**	**297**	**(29.7)**	**75**	**313**	**(24.0)**	**25-44**	**172**	**(14.5-25.6)**	**189-208**	**782**	**(24.2-26.6)**

^a^ cannot be exactly provided due to varying numbers (up to 5) of nymphs in the pools; The range of infection rate is the minimal and maximal infection rate (MinIR, MaxIR), assuming that either only one or all of the nymphs in a pool were positive.

pos = positive.

### Co-infections

The animals, host-attached and questing ticks have been investigated for *A. phagocytophilum*, *Babesia* spp. and *Rickettsia* spp. (questing ticks only) in previous studies [24,25]. The following double infections with CNM were observed (for nymphs, only those investigated individually were considered for co-infections, resulting in a total of 101 nymphs): (i) with *B. microti* in 11 ticks (7 males, 4 females; 1 in the Saarland; 10 in Leipzig), (ii) with *B. divergens* in 2 ticks in Bavaria (1 male, 1 female), (iii) with *Babesia* sp. EU1 in 1 male tick in Leipzig, (iv) with *A. phagocytophilum* in 22 ticks (10 males, 10 females, 2 nymphs; 4 in Bavaria; 5 in the Saarland; 13 in Leipzig), (v) with *R. helvetica* in 16 ticks (10 males, 6 females; 7 in the Saarland; 9 in Leipzig), (vi) with *Rickettsia* spp. in one further female *I. ricinus* in the Saarland. Six ticks (5 males, 1 female) had a triple infection with CNM, *A. phagocytophilum* and *R. helvetica* (4 in the Saarland and 2 from Leipzig). The statistical analysis of co-infection taking into account both stage and sex of the tick as well as collection site, was performed for double infections with CNM (all co-infections in general, and CNM with *Rickettsia* spp., *Babesia* spp. and *A. phagocytophilum*, respectively). For the purpose of this analysis, triple infected ticks were counted as double infected with each of the pathogens. No statistically significant effects of stage and sex regarding co-infections of any type could be found.

## Discussion

In this study, we investigated small mammals, their ticks and questing *I. ricinus* for the occurrence of DNA of CNM in areas with sympatric *I. ricinus* and *D. reticulatus* populations. Rodents have been found to harbour CNM and this group of animals has thus been suggested as reservoir hosts [12-14,28-31]. The high infection rates of yellow-necked mice and bank voles in the present study corroborate this hypothesis. DNA of CNM was also detected in a striped field mouse, but the number of animals (n=3) was too low to draw a conclusion from this finding. We found the highest percentage of infected animals in August, when the developmental stages of both *I. ricinus* and *D. reticulatus* are most active [4,32]. Rodents were still infected in October and November, but DNA was not detected in the animals in spring. Hence, it may be that the rodents cannot carry the infection over the winter. All the same, the number of animals caught in spring was low, and to prove this hypothesis, more systematic studies with a larger number of animals are needed.

We compared different organs as material for detection of DNA of CNM in epidemiological studies. During experimental infection of rats, infection was not observed after 15 days in blood, spleen and liver, weak infection in the spleen after 30 days, and infection in all three sample types after 60 days. Histologically, the agent was recognized in the spleen sinus endothelial cells forming intracellular inclusions in the cytoplasm. Consequently, blood does not seem to be an ideal target material, because infection may be detectable only after more than 30 days, assuming the course of infection in mice is similar to the one observed in rats. Experimentally infected mice did not have detectable CNM-DNA in their spleen after 10 days [12]. Our finding that blood samples were negative when other organs were positive may therefore reflect different time points of infection [12], but further experimental infections of mice are lacking thus far. According to our own results and a comparison with experimental infection in the literature, we conclude that spleen and kidney are the best organ material for detection of DNA of CNM in epidemiological studies involving rodents. Skin seems unsuitable for the detection of DNA of CNM. At any rate, all results taken together strongly argue for a systemic course of infection [12,13], which is supported by observations in human patients. Fehr *et al.*[18] and von Loewenich *et al.*[20] detected DNA of CNM in peripheral blood samples of human patients. Peková *et al.*[19] were able to show the agent in patients’ granulocytes in transmission electron microscopy. In spite of this, the agent has as yet not been observed in blood smears.

In comparison to previous studies from other areas in Europe, the prevalence rates in the small rodents in the present study were high and similar to the initial investigations in Japan where 7 out of 15 wild caught Norway rats were infected [12]. In Sweden, between 4 and 10% of rodents (bank voles, field voles (*Microtus agrestis*), wood mice (*A. sylvaticus*) and yellow-necked mice), but no shrews were infected. In different sites in Sweden the prevalence ranged from 0% to 12.5% [29]. The fact that blood was used in that study may account for the lower prevalence compared to our study. In the Netherlands, spleens were investigated and prevalence was higher: 21.7% of wood mice, 25% of common voles, and 11.4% of bank voles, but none of the two yellow-necked mice were infected. Shrews were not infected either [13]. Our findings strongly support the hypothesis that rodents may be competent reservoir hosts and that they may play an important role in the endemic cycle of CNM [29]. In line with the findings in Sweden and the Netherlands, we did not detect CNM in any insectivore species [13,29]. With a prevalence about twice as high as in questing ticks a reservoir function of the rodents seems highly likely at least for the season of the year when ticks are active.

Altogether 6.5% of host-attached *I. ricinus* ticks (larvae and nymphs) were positive, which is lower in comparison to questing ticks. When interpreting this result, it has to be taken into account that the questing ticks and the rodents and their ticks were from different years (2009: questing ticks; 2010/11: rodents and their ticks). Tick-host-pathogen cycles are influenced by a lot of factors, many of which are still largely unknown and may account for differences in prevalence rates at different time points. Variations have, for example, been shown in the prevalence over several years with *A. phagocytophilum* in ticks [33].

Previously investigated questing *D. reticulatus* collected from vegetation had no detectable DNA of CNM in them [13,34]. All positive developmental stages of *D. reticulatus* from the present study were from bank voles that were positive for CNM in the blood; therefore it may be assumed that the detection of CNM-DNA represents the blood meal. The same can be said for the *I. ricinus* larvae from the small mammals, as they were all engorged. It is not known yet whether transovarial transmission occurs, but 55 larval pools from questing larvae were negative [13]. Unfortunately, we did not have questing larvae available for the present study. It seems likely that CNM cannot be transmitted transovarially and experimental evidence for transstadial transmission is also lacking, thus, an experimental setting to uncover the epidemiological transmission cycle is needed.

Average prevalences of CNM in questing *I. ricinus* or *I. persulcatus* ticks in several Eurasian countries range from 0% to 16.7% [9,13,14,22,23,35-42]. Thereby, the prevalence rates in *I. ricinus* seem to average around 6%, whereas the prevalence in *I. persulcatus* seems to be lower, reaching up to 3.8%, but staying in general around or below 1% [23,30,43]. Most previous studies used conventional PCR; sometimes combined with sequencing and/or hybridization with oligonucleotide probes for species identification. The increased sensitivity of real-time PCR used in the present and another study may also account for the increased detection rate of DNA of CNM in these studies [13,23]. In our investigated area, the rodents and ticks seem to provide a very efficient system for CNM to thrive on and to develop such high prevalence. Whether this persists over time needs further systematic and longitudinal investigations in both ticks and host species.

Co-infections of CNM in ticks in our study with *R. helvetica*, *A. phagocytophilum* and *B. microti* may be explained by using the same (suggested) reservoir host animals [3,44,45]. No difference was found in prevalence between males and females in infection rates, which is in line with our findings [13]. Data from northern Italy found females significantly more often co-infected with more than one pathogen [36], whereas in our study, there was a tendency in the opposite direction comparing female ticks with male ticks.

## Conclusion

The high prevalence of CNM in this study in metropolitan and recreational areas points towards the necessity for a larger scale surveillance of risk populations and/or humans after being bitten by ticks, in order to get a picture of the full scale public health impact. Especially among immunocompromised patients with fever of unknown origin this may be largely underdiagnosed. First and foremost, isolation of the pathogen should now be attempted in order to develop diagnostic tools such as specific serological tests and to study the transmission cycle and pathogenic properties of the agent in experimental and controlled settings.

## Competing interests

The authors declare that they have no competing interests.

## Authors’ contributions

CS and MP conceived the idea and design of the study. CS, DW and MP carried out field and laboratory work. MM performed the statistical analysis. CS analysed the data and wrote the manuscript. DW, MP and KP critically revised the manuscript. All authors read and approved the final version of the manuscript.
